# 42.4 THE ENDOCANNABINOID SYSTEM IN FIRST-EPISODE PSYCHOSIS

**DOI:** 10.1093/schbul/sby014.177

**Published:** 2018-04-01

**Authors:** Jarmo Hietala

**Affiliations:** University of Turku

## Abstract

**Background:**

The endocannabinoid (EC) system comprises of fatty acid neurotransmitters such as anandamide and 2-acylglycerol, at least two specific cannabinoid (CB) receptor subtypes and enzyme machinery for synthesis and degradation of ECs. The EC system regulates a wide array of functions in central nervous system and peripheral tissues such as brain development, plasticity, reward and stress sensitivity as well as metabolic functions a such as energy/lipid metabolism including liver function and insulin resistance (1). One of the aims of the METSY project (www.metsy.eu) is to study the central and peripheral EC system in FEPs and their involvement in brain mechanisms in psychosis as well as related metabolic co-morbidities. The cannabinoid CB1 receptor (CB1R) is the most abundant cannabinoid receptor subtype in the human brain and is the predominant mediator of various EC effects. In this first series of METSY experiments we report in vivo brain CB1R binding characteristics in patients with first-episode psychosis (FEP) and controls.

**Methods:**

Brain CB1R availability was measured using [18F]-FMPEP-d2 and 3D ECAT HRRT positron emission tomography (PET) using distribution volume (DVt) as a proxy for CB1R availability. Our studies in healthy volunteers revealed a marked sex difference in [18F]-FMPEP DVt with males having higher CB1R availability. The overall CB1R sex difference was widespread but also regionally different with most significant effects in the occipital cortex. Therefore, subsequent studies focused first on male patients with FEP and controls (n=18).

**Results:**

The results indicate a significant and relatively widespread decrease in CB1R DVt in patients with FEP in fronto-temporal regions, putamen, posterior cingulate as well as parietal regions with large effect sizes. Largely similar results were seen in an independent sample of medication-free male patients with FEP (n=17, the other METSY PET site, King’s College, London, UK) (2). Those studies used another validated CB1R tracer, the [11C]-MePPEP. The reduced CB1R in patients was also state-dependent as [18F]-FMPEP DVt correlated negatively with psychotic symptoms (BPRS) in a highly significant manner.

**Discussion:**

Epidemiological studies have convincingly shown that cannabis use is associated with an increased risk of both psychotic symptoms and schizophrenia-like psychoses. We now provide direct in vivo evidence for a robust and partly state-dependent dysregulation of the endocannabinoid system in first-episode psychosis. This is well in line with a recent PET study with CB1R tracer, [11C]-OMAR in patients with schizophrenia (3). Based on previous experimental data the reduced CB1R availability is indicative of an increased endocannabinoid drive in psychosis.

**Acknowledgements:**

Funding from the EU’s 7th Framework Programme; METSY - Neuroimaging platform for characterization of metabolic co-morbidities in psychotic disorders (no. 602478).

**References:**

1) Eriksson O et al The Cannabinoid Receptor-1 Is an Imaging Biomarker of Brown Adipose Tissue. J Nucl Med. 2015 Dec;56(12):1937–41.

2) METSY: Endocannabinoid pathways in early psychosis – brain imaging studies (Hietala J, Howes O, Laurikainen H, Borgan F and the METSY PET Imaging group).

3) Ranganathan M et al Reduced Brain Cannabinoid Receptor Availability in Schizophrenia. Biol Psychiatry. 2016 Jun 15;79(12):997–1005.

